# Utilization of non-pneumatic anti-shock garment for the management of obstetric hemorrhage among healthcare providers in north Shewa zone, Ethiopia

**DOI:** 10.3389/fpubh.2023.1052885

**Published:** 2023-04-27

**Authors:** Birhan Tsegaw Taye, Mulualem Silesh Zerihun, Tebabere Moltot Kitaw, Fetene Kasahun Amogne, Geremew Kindie Behulu, Tesfanesh Lemma Demisse, Moges Sisay Chekole, Girma Wogie Fitie, Solomon Adanew Worku, Desta Mekete Kibiret, Addisu Andualem Ferede, Kalkidan Bejtual, Temesgen Desalegn, Agumas Eskezia Tiguh, Muhabaw Shumye Mihret, Azmeraw Ambachew Kebede

**Affiliations:** ^1^School of Nursing and Midwifery, Asrat Woldeyes Health Science Campus, Debre Berhan University, Debre Birhan, Ethiopia; ^2^Department of Midwifery, Faculty of Public Health and Medical Science, Mettu University, Metu, Ethiopia; ^3^Department of Midwifery, College of Medicine and Health Science, Debre Markos University, Debre Markos, Ethiopia; ^4^Department of Midwifery, Bule Hora University, Bule Hora, Ethiopia; ^5^Department of Midwifery, Jigjiga University, Jigjiga, Ethiopia; ^6^Department of Clinical Midwifery, School of Midwifery, College of Medicine and Health Sciences, University of Gondar, Gondar, Ethiopia

**Keywords:** healthcare provider, NASG, obstetrics hemorrhage, Ethiopia, women

## Abstract

**Background:**

Global maternal deaths have either increased or stagnated tragically. Obstetric hemorrhage (OH) remains the major cause of maternal deaths. Non-Pneumatic Anti-Shock Garment (NASG) has several positive results in the management of obstetric hemorrhage in resource-limited settings where getting definitive treatments are difficult and limited. Therefore, this study aimed to assess the proportion and factors associated with the utilization of NASG for the management of obstetric hemorrhage among healthcare providers in the North Shewa zone, Ethiopia.

**Methods:**

A cross-sectional study was conducted at health facilities of the north Shewa zone, Ethiopia from June 10th-30th/2021. A simple random sampling (SRS) technique was employed among 360 healthcare providers. Data were collected using a pretested self-administered questionnaire. EpiData version 4.6 and SPSS 25 were used for data entry and analysis, respectively. Binary logistic regression analyses were undertaken to identify associated factors with the outcome variable. The level of significance was decided at a value of *p* of <0.05.

**Results:**

The utilization of NASG for the management of obstetric hemorrhage among healthcare providers was 39% (95%CI: 34–45). Healthcare providers who received training on NASG (AOR = 3.3; 95%CI: 1.46−7.48), availability of NASG in the health facility (AOR = 9.17; 95%CI: 5.10–16.46), diploma (AOR = 2.63; 95%CI: 1.39–3.68), bachelor degree (AOR = 7.89; 95%CI: 3.1–16.29) and those healthcare providers who have a positive attitude toward utilization of NASG (AOR = 1.63; 95%CI: 1.14–2.82) were variables positively associated with the utilization of NASG.

**Conclusion:**

In this study, almost two-fifths of healthcare providers used NASG for the management of obstetrics hemorrhage. Arranging educational opportunities and continuous professional development training for healthcare providers, providing in-service and refresher training, and making it available at health facilities may help healthcare providers to effectively use the device, thereby reducing maternal morbidity and mortality.

## Introduction

Global statistics show that more than 303,000 women die annually and 2.6 million babies were stillborn, half occurring during the third trimester (1) with the vast majority (94%) of these deaths occurring in low resource settings (2). From the global figure, approximately 14,000 maternal deaths occur each year in Ethiopia with a higher lifetime risk of maternal mortality (3). The current evidence shows that four women die from every 1,000 live births in Ethiopia, which is the highest maternal death in the world (4).

Obstetric hemorrhage (OH) remains one of the foremost contributing factors to maternal mortality (5), accounting for 25 percent of maternal deaths globally (5, 6). OH is an emergency in which a woman loses a very large amount of blood during pregnancy, during delivery, and after childbirth. An estimated, 150,000 women die from a preventable obstetric hemorrhage each year; most of these deaths occur in low-resource settings (7, 8). According to the World Health Organization (WHO) report almost all maternal deaths occurred in low-income and lower-middle-income countries, and two-thirds (65%) occurred in the African Region (3).

One of the effective and recently recommended first-aid devices for the management and prevention of OH is the use of a non-pneumatic anti-shock garment (NASG) (9). The NASG is a valuable innovation for reducing maternal mortality and morbidity in developing nations (10, 11). It is a lightweight, reusable garment made of neoprene and Velcro, containing five segments that are closed tightly around the legs, pelvis, and abdomen. It also applies pressure to decrease blood flow to and within the compressed area, and redirects blood to the vital organs (i.e., heart, lungs, and brain), thereby stabilizing vital signs and resolving hypovolemic shock (12). It has been demonstrated that NASG reduces mortality for women in shock at the time of referral (13, 14). A systematic review also reported that NASG reduces maternal death due to OH compared the non-users (15). Similarly, a global health study showed that NASG reduces blood loss by 42–55%, maternal morbidity by 44–66%, mortality by 34–66%, and the need for emergency hysterectomies by 45–56% (16). About 96% of women survive following the use of NASG for severe OH. NASG reduces financial losses, particularly the costs of transfusions and medical care (17). The WHO and International Federation of Obstetricians and Gynecologists (FIGO) recommended the use of NASG for the prevention and treatment of postpartum hemorrhage (PPH) (18, 19).

Investing in women’s health, including ensuring women have safe and healthy pregnancies, deliveries, and post-partum periods contributes not only to improving household health outcomes but also advances social and political developments for the country (20, 21). Early NASG application in primary health care for women in hypovolemic shock has the potential to be cost-effective across many clinical settings (22). Many women in low-resource settings deliver far from health institutions. Thus, once bleeding starts, these women die before reaching or receiving appropriate treatment. WHO recommends NASG as a temporary measure to regain hemodynamic stability and allow patient transfer for definitive PPH treatment (23). However, for women in mild shock, NASG has smaller and uncertain effects (17).

Despite the efficacy of NASG in reducing maternal morbidity and mortality, its utilization remains low. In Nigeria, 46% of midwives use NASG for the management of PPH (24). Similarly, in Jimma Ethiopia, only 36.2% of midwives use NASG for the management of PPH and only 54% of the midwives had good knowledge of NASG (13). Evidence addressing healthcare providers (HCPs) utilization of NASG for the management of OH in Ethiopia is not well-documented and challenges the healthcare system. Understanding the factors responsible for the non-utilization of NASG will give insight into probable interventions that might improve utilization. Therefore, this study aimed to assess the proportion of NASG utilization and its associated factors among HCPs in the North Shewa zone, Ethiopia.

## Methods

### Study design, setting, and period

This cross-sectional study was conducted at health facilities in the North Shewa zone, Amhara regional state, northeast Ethiopia from June 10th–30th/2021. Amhara regional state has 11 zones; of these, North Shewa is one of the zones in the Amhara regional state. Debre Birhan is the capital city of the North Shewa Zone and is located 130 km Northeast of Addis Ababa on the Ethiopian highway. The zone comprises 439 kebeles and 24 districts with an estimated total population of 2,335,205 with 1,138,737 females and 1,196,408 males. There are 95 health centers, 13 Hospitals (of which two are private primary hospitals) 10 primary, two general, and one comprehensive specialized hospital. There are 772 Nurses, 452 midwives, 120 Laboratory professionals, 130 Pharmacists, 219 General Practitioners, 15 Obstetric specialists, 54 integrated emergency surgical officers (IESO), 56 Anesthesia, 22 Radiology technicians, and 15 mental health professionals working in the zone. Therefore, the study was conducted at five public hospitals and seven health centers in the North Shewa zone. The named health institutions were Debre Berhan comprehensive specialized hospital, Enat hospital, Shewarobit hospital, Arerti hospital, Ataye hospital, Enewari health center, Shewarobit health center, Ataye health center, Alem ketema health center, Debresina health center, Arerti health center, and Ayer tenna health center.

### Source and study population

All obstetric healthcare providers who were working in North Shewa Zone public Health facilities were the source of population. And all obstetric care providers who are working in the selected public healthcare facilities and fulfilled the selection were the study population.

### Eligibility criteria

#### Inclusion criteria and exclusion criteria

All obstetric care providers who were staffed in the respective wards in each public health facility were included in this study, whereas obstetric care providers who had been on leave at the time of data collection were excluded.

### Sample size determination

The sample size for this study was determined by using a single population proportion formula by considering the following assumptions: 95% level of confidence, 36.2% provider’s utilization of NASG for PPH from a previous study (13), and 5% margin of error.


$$n=\frac{{\left(Z\alpha /2\right)}^{2}p\left(1-p\right)}{d^{2}}=\frac{{\left(1.96\right)}^{2*}0.362\;\left(0.638\right)}{\left(0.05\right)2}=355$$

Taking a non-response rate of 5%, a total of 373 study participants was obtained.

### Sampling technique and procedure

**Figure 1:**
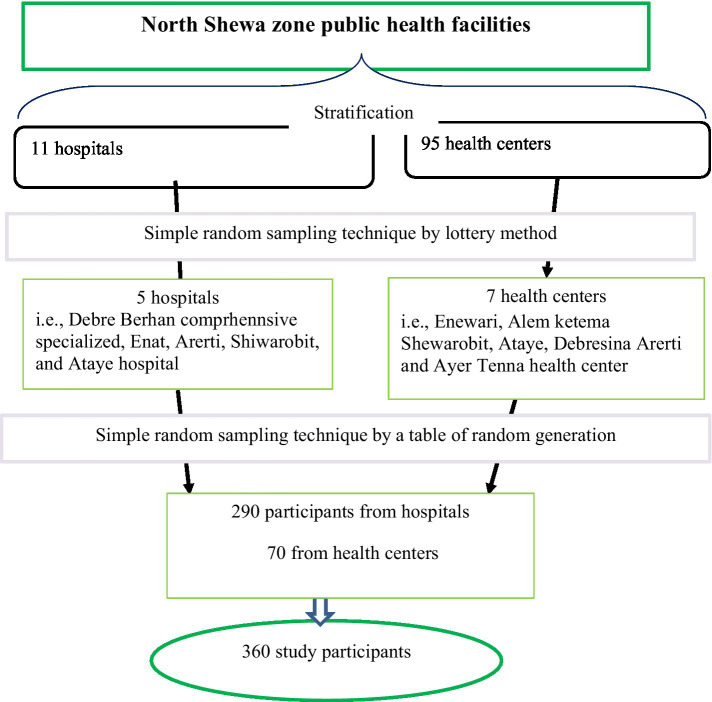
Utilization of non-pneumatic anti-shock garment for the management of obstetric hemorrhage among healthcare providers in north Shewa zone, Ethiopia.

Data were collected from 12 randomly selected health facilities (5 public hospitals, and 7 health centers). A sampling frame was prepared by obtaining the list of obstetric care providers from each health facility’s human resources office. The total number of health professionals in the study setting during the data collection period was 1,215 and 466 in the selected facilities. Then, the total sample size was allocated to each selected health facility proportionally. Finally, SRS technique was employed to choose the study participants using a table of random generation. Thus, Debre Berhan comprehensive specialized hospital (*n* = 116), Enat hospital (*n* = 73), Arerti hospital (*n* = 63), Shewarobit hospital (*n* = 61), Ataye hospital (*n* = 58), Enewari health center (*n* = 18), Shewarobit health center (*n* = 13), Ataye health center (*n* = 15), Alem ketema health center (*n* = 11), Debresina health center (*n* = 12), Arerti health center (*n* = 12); and Ayer Tenna health center (*n* = 14) were included in the study (Figure 1).

### Measurements and operational definitions

The dependent variable in this study was HCPs’ uptake of NASG. Accordingly, the participants were asked about their utilization of NASG in the management of obstetric hemorrhage at least once, the possible answers were Yes or No. A score of “1” was given for Yes and a score of “0” was given for No (13, 25).

#### Knowledge toward NASG

The study participants were asked 11 questions and those who scored 50% and above from all knowledge questions were considered as knowledgeable while those who below 50% were graded as having poor knowledge. Likewise, Attitude toward NASG refers to the extent to which an individual has a positive or negative estimation of the behavior of interest. HCPs’ attitude was measured using 10 questions: Each question has five points Likert scale (1 = strongly disagree, 2 = disagree, 3 = neutral, 4 = agree, 5 = strongly agree). The total score ranged from 10 to 50, attitude was considered “Positive” if the percentage score was 50% and above and “Negative” if less than 50% (13).

#### Educational level

In the Ethiopian context, a diploma means (grades 10 + 3 years of college or 12 + 3 years of college and a bachelor’s degree 12 + 3 or 4 years of university study).

### Data collection tools, procedures, and quality control

The data collection tool was developed by reviewing literature (13, 26, 27) and data were collected using a structured self-readministered questionnaire. Initially, the questionnaire was prepared in English and translated into Amharic language and back to English to ensure its consistency and better understandability. Expert researchers judged the suitability of the questionnaire and the overall standardized alpha coefficient was used to measure the reliability or internal consistency of the tool, which became 0.86. The questionnaire comprises socio-demographic characteristics, professional and work-related characteristics, and questions assessing the healthcare provider’s knowledge of NASG and attitude toward its utilization.

Before the actual data collection, a pretest was done on 5% of the calculated sample size at Deneba hospital and appropriate corrections were made such as the logical order of some questions, and some words difficult to understand were revised. The two-day training was given on the overall data collection process and safety measures during the data collection. A total of 24 individuals have participated in the data collection and supervision process such as 13 Diploma, 6 BSc, and 4 MSc midwives. During data collection, the questionnaire was checked for completeness by the supervisors and the principal investigator.

### Data processing and analysis

Data entry was carried out using EpiData version 4.6 and analyzed using SPSS version 25. Frequency tables and graphs were used to present descriptive and analytic statistics of participants. The multicollinearity assumption was checked and it was acceptable with a variance inflation factor of <10. The binary logistic regression (i.e., bivariable and multivariable) model was fitted to identify independent predictors, and variables having a value of *p* of less than 0.25 were included in the multivariable logistic regression analysis. In the multivariable logistic regression, a value of *p* of <0.05 with 95% CI for the AOR was used to determine the significant association of variables.

### Ethical approval

The study was conducted under the Ethiopian Health Research Ethics Guideline and the declaration of Helsinki. Ethical clearance was obtained from the Institutional Ethical Review Board (IRB) of Debre Berhan University (protocol number: p014 and approval reference number: RCSCH 01/166/2013). A formal letter of administrative approval was gained from each selected hospital. Written informed consent was taken from each of the study participants after a clear explanation of the aim of the study and that participation was voluntary. Moreover, there are no individually detailed data, videos, or images.

## Results

### Socio-demographic characteristics

In this study, a total of 360 healthcare workers were involved, making a 96.5% response rate. The median age of the study participants was 28 years (ranging from 23 to 41). Of the study participants, 217 (60.3%) were within the age group of 25–29 years, 223 (61.9%) were male, and 326 (90.6%) were Amhara in ethnicity (Table 1).

**Table 1:** Socio-demographic characteristics of study participants among healthcare providers in north Shew Shewa zone health facilities, 2021 (*n* = 360).

Characteristics	Category	Frequency	Percentage (%)
Age in year	<25	28	7.8
	25–29	217	60.3
	30–34	99	27.5
	> = 35	16	4.4
Sex	Female	38.1	38.1
	Male	61.9	61.9
Current marital status	Single	184	51.1
	Married	172	47.8
	Divorce	2	0.6
	Separated	2	0.6
Religion	Orthodox	338	93.9
	Muslim	6	1.7
	Protestant	16	4.4
Ethnicity	Amhara	326	90.6
	Oromo	20	5.6
	Others@	14	3.9
Source of information about NASG	Training	52	14.49
	Health institution	157	43.59
	College/university	137	38.1
	Internet	66	18.3

@, Tigre, Gurage, and Argoba.

### Workplace and profession-related characteristics

Among the participants, 290 (80.6%) were working from hospitals and 261 (72.5%) of them reported that they had working experience of 1–5 years. Three hundred and eight (85.6%) of HCPs did not receive training on NASG and 253 (70.3%) were Midwives by profession. Two third of the study participants (66.9%) had a bachelor’s degree (Table 2).

**Table 2:** Working place and profession-related characteristics among healthcare providers in north Shew Shewa zone health facilities, 2021 (*n* = 360).

Characteristics	Category	Frequency	Percentage (%)
Facility type	Hospital	290	80.6
	Health center	70	19.4
Profession	Midwifery	253	70.3
	Medical doctor	75	20.8
	IESO	27	7.5
	Health officer	5	1.4
Professional qualification	Diploma	86	23.9
	Bachelor degree	241	66.9
	Master’s degree	28	7.8
	Obstetric specialist	5	1.4
Professional working experience in years	1–5	261	72.5
	6–10	78	21.7
	>10	21	5.8
Availability of NASG in the facility	Yes	191	53.1
	No	169	46.9
Received NASG training	Yes	52	14.4
	No	308	85.6
Intention to use NASG	Yes	257	71.4
	No	103	28.6

NASG, non-pneumatic anti-shock garment.

### HCPs’ knowledge of non-pneumatic anti-shock garment

In this study, 59.2% of respondents were knowledgeable about the NASG while 40.8% had poor knowledge of NASG.

### Factors associated with the utilization of non-pneumatic anti-shock garment for obstetric hemorrhage management

Multivariable logistic regression analysis showed that HCPs who were; had the professional qualification of diploma and bachelor’s degree, received training on NASG, had availability of NASG in the health facility, and had a positive attitude toward utilization of NASG were found to be significantly associated with HCPs’ utilization of NASG.

**Figure 2:**
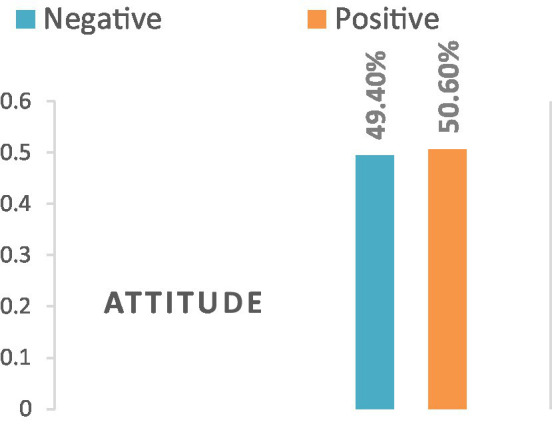
Distribution of healthcare providers’ attitude toward non-pneumatic anti-shock garment utilization in north Shew zone health facilities, 2021.

HCPs with diploma and bachelor degree holders were 2.63 and 7.89 times more likely to be utilized NASG than senior obstetricians (AOR = 2.63; 95% CI: 1.39, 3.68) and (AOR = 7.89; 95% CI: 3.1, 16.29) respectively. Similarly, HCPs who received NASG training had 3.3 times more odds of the utilization of NASG than their counterparts (AOR = 3.3; 95% CI: 1.46, 7.48). Moreover, the odds of NASG utilization were 9.17 times higher among HCPs who were working in facilities where NASG is available as compared with their counterparts (AOR = 9.17; 95% CI: 5.10, 16.46). The likelihood of NASG utilization for OH was 1.63 times higher among HCPs who had a positive attitude compared with that of those who did not do so (AOR = 1.63; 95% CI: 1.14, 2.82) (Figure 2; Table 3).

**Table 3:** Logistic regression analysis of factors associated with utilization of NASG for OH management among health care providers in North Shewa zone (*n* = 360).

Variables	Utilized NASG		COR (95% CI)	AOR (95% CI)
	Yes	No		
Age in years				
<25	15	13	0.49 (0.22, 1.08)	0.17 (0.06, 1.49)
25–29	78	139	0.54 (0.23, 1.26)	0.63 (0.49, 2.82)
30–34	38	61	1.91 (0.52, 6.94)	1.14 (0.02, 1.17)
> = 35	11	5	1	1
Sex				
Female	61	76	1.41 (0.91, 2.17)	1.51 (0.85, 2.66)
Male	81	142	1	1
Current marital status				
Single	63	125	1	1
Married	79	93	1.69 (1.10, 2.58)	1.4 (0.76, 2.56)
Professional qualification				
Diploma	19	67	2.46 (1.39, 4.35)	2.63 (1.27, 5.46)*
Bachelor degree	99	142	10.58 (3.91, 18.63)	7.89 (3.1, 16.29)**
Master’s degree	21	7	5.29 (0.82, 33.99)	6.98 (0.57, 85.88)
Obstetric specialist	3	2	1	1
Facility type				
Hospital	128	162	1	1
Health center	14	56	0.32 (0.17, 0.59)	1.4 (0.76, 2.56)
Professional working experience in years				
01-May	98	163	1	1
06-October	38	40	1.58 (0.95 2.63)	2.02 (0.95, 4.29)
>10	6	15	0.67 (0.25, 1.77)	0.18 (0.05, 12.23)
Received NASG training				
Yes	35	17	3.87 (2.07, 7.23)	3.3 (1.46, 7.48)**
No	107	201	1	1
Availability of NASG in the health facility				
Yes	118	73	9.77 (5.8, 16.45)	9.17 (5.10, 16.46)*
No	24	145	1	1
Knowledge				
Poor knowledge	50	97	1	1
Knowledgeable	92	121	1.48 (0.95, 2.28)	1.11 (0.62, 1.98)
Attitude				
Negative	60	118	1	1
Positive	82	100	1.61 (1.05, 2.47)	1.63 (1.14, 2.82)**

^*^p < 0.05, ^**^p < 0.001.

## Discussion

This study was conducted to assess the Utilization of Non-Pneumatic Anti-Shock Garment for the management of obstetric hemorrhage among healthcare providers in North Shewa, Ethiopia, 2021. Thus, this study revealed that the proportion of NASG utilization among healthcare providers was 39% with a 95% CI of (34–45). Besides, healthcare providers having professional qualifications of diploma and bachelor degree significantly associated factors with the utilization of NASG, who have received g training on NASG, availability of NASG in the facility, and having a positive attitude toward NASG were the variables significantly associated with the outcome variable.

The finding of this study is in line with the study conducted in Jimma zone, Ethiopia-36.2% (13) and Nigeria 35% (25). However, it is lower than the study from southern Ethiopia, 48.5% (26), Niger-46.4% (28), and Nigeria-73.7% (29). This could be due to variations in training and NASG availability and knowledge and attitude of healthcare providers. In this study, only 59.2% and nearly half (49.4%) of healthcare providers had adequate knowledge and unfavorable attitude, respectively. Besides, the unavailability of NASG in the health facility (46.9%) may contribute to the non-utilization and calls concerning bodies regarding the need for refresher training and availing the device in each health facility. In addition, the differences might be attributed to that the previous studies (26, 28, 29) were merely concerned with PPH management, but our study included the management of other obstetric hemorrhages.

This study indicated that HCPs who received training on NASG had 3.3 times more odds of the utilization of NASG than their counterparts. This may be because attending training can help healthcare providers to foster basic knowledge and skills on how to apply, and remove NASG and this can improve their utilization of non-pneumatic anti-shock garments. It was consistent with studies from the Jimma zone, Ethiopia (13), and southern Ethiopia (26) which showed that training could increase the likelihood of NASG utilization. Therefore, health professionals receiving NASG training are likely to have more knowledge and skills about the utilization of NASG for the management of OH, thereby adhering to the respective guidelines as compared to non-trained healthcare providers (13).

On the other hand, the likelihood of NASG utilization for OH was 1.63 times higher among HCPs who had a positive attitude compared with those who had a negative attitude. It was consistent with the study from Ethiopia (13). This might be explained by a positive attitude being one of the most important things that can lead to achieving the desired level of professional success. Similarly, this study revealed that the odds of NASG utilization were 9.17 times higher among HCPs who were working in facilities where NASG is available as compared with their counterparts. This might be due to the availability of NASG exposing the health care professionals to perform their acquired skills, as regular usage should make them familiar with it. This was also supplemented with the study from Jimma public hospitals (13). Besides, a study from Sokoto State Nigeria showed the non-availability of NASG plays on perceived hindrances to the utilization of this anti-shock garment (30).

### Implication for practice

In this study findings, as gaps were found in the knowledge and attitude of HCPs on NASG, this calls for refresher training for HCPs on the usage of NASG. It is hoped that this will also improve the attitude of providers toward the equipment. In a bid to improve utilization of the low-cost technology equipment, a monitoring system is hereby recommended as this might help to improve utilization. Correspondingly, a large number of the respondents reported the non-availability of the NASG as a factor associated with non-usage, the hospital authority should ensure that all maternity units have access to the NASG. This result calls for stakeholders like the Ethiopian Midwife association (EMwA) and local health facilities to ensure the availability of NASG in health facilities and educational programs.

### Strength and limitations

The authors would like to acknowledge some of the limitations of the current study. First, the cross-sectional nature of the study design might not be a guarantee to infer causality between utilization and the suggested explanatory variables. Finally, we did not get enough related articles to compare our results which makes our discussion shallow. Despite the above-mentioned limitations, this study sought to assess rarely addressed issues of OH and was able to provide important information to healthcare stakeholders.

## Conclusion

In this study, almost two-fifths of healthcare providers used NASG for the management of obstetrics hemorrhage. Having professional qualifications of a diploma and bachelor’s degree, receiving training on NASG, its availability in the health facility, and a positive attitude toward utilization were positively associated factors with the utilization of NASG for OH management. The healthcare professionals who work in maternity units ought to get the training and handy equipment that may encourage the sustainable utilization of the garment.

## Data availability statement

The original contributions presented in the study are included in the article/Supplementary material, further inquiries can be directed to the corresponding author.

## Ethics statement

The studies involving human participants were reviewed and approved by the Debre Berhan University. The patients/participants provided their written informed consent to participate in this study.

## Author contributions

BT had a major role in the conceptualization, data curation, develop the proposal, and data entry. All authors participate in the formal analysis and writing the original draft, equally participated in manuscript preparation and revision, and approved the manuscript to be considered for publication.

## Conflict of interest

The authors declare that the research was conducted in the absence of any commercial or financial relationships that could be construed as a potential conflict of interest.

## Publisher’s note

All claims expressed in this article are solely those of the authors and do not necessarily represent those of their affiliated organizations, or those of the publisher, the editors and the reviewers. Any product that may be evaluated in this article, or claim that may be made by its manufacturer, is not guaranteed or endorsed by the publisher.

## Abbreviations

NASG: Non-Pneumatic Anti-Shock Garment
HCP: Healthcare Provider
OH: Obstetric Hemorrhage
PPH: Postpartum Hemorrhage
WHO: World Health Organization
