# Supplementary material for: Utilization of non-pneumatic anti-shock garment for the management of obstetric hemorrhage among healthcare providers in north Shewa zone, Ethiopia
Source: Front Public Health. 2023 Apr 27;11:1052885. doi: 10.3389/fpubh.2023.1052885 (PMC10173860; doi:10.3389/fpubh.2023.1052885)
Supplement: Supplementary file 1 [file Table_1.DOCX]

**English version Questionnaire**

Health facility category (**tick** **√)** Hospital Health centre

Questionnaire identification number___________ Date of data collection ___________E.C

Introduction: How are you? My name is ____________________, I am a data collector for the

research entitled of ‘‘Utilization of Non-Pneumatic Anti-Shock Garment for Obstetric haemorrhage management among healthcare providers in North Shewa zone, Ethiopia**”.** You are selected to be one of the Participants in the study. Your response will provide valuable information about the issue under this study.

**Person to contact**: Birhan Tsegaw

Phone No: +251912671560 Email: [tsegawbirhan2@gmail.com](tsegawbirhan2@gmail.com%20)

Are you willing to participate in this study? **(tick as √)**

1. Yes Sign and proceed to the questionnaire
2. No
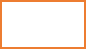
 Thank you!!!

Signature of the respondent -----------------------

| **Part-I Socio-demographic characteristics of participants** | | | |
| --- | --- | --- | --- |
| Code | Questions | Possible responses | Skip to |
| 101. | How old are you? | _________years |  |
| 102. | Sex of respondent? | 1. Female 2. Male |  |
| 103. | What is your marital status? | 1. Single 2. Widowed 3. Divorced 4. Separated 5. Married |  |
| 104. | What is your religion? | 1. Orthodox 2. Muslim 3. Protestant Other(specify)__________ |  |
| 105. | What is your ethnicity? | 1. Amhara 2. Oromo 3. Tigray Other (Specify)_________ |  |
| 106. | What is your professional qualification? | 1. Diploma midwife 2. BSc midwife 3. General practitioner 4. Emergency surgeon 5. Gyn/obs specialist Other (Specify)_______ |  |
| 107. | How many years of experience do you have as health care professional? | ____________years |  |
| **Part- II Knowledge on Non-pneumatic Anti-Shock Garment (NASG)** | | | |
| 201. | Have you ever heard about Non pneumatic Anti-Shock Garment (NASG)? | 1. Yes 2. No |  |
| 202. | What is the NASG looks like? | 1. Bottom half of suit 2. Gown 3. Trouser |  |
| 203. | What is your first source of information about NASG? | 1. Training 2. Health institution as they used management modality 3. College/ university education 4. Internet Other specify_________ |  |
| 204. | How many segments does NASG have? | 1. Four 2. Six 3. Eight 4. Nine |  |
| 205. | What is/are the function of NASG? (more than one answer is possible) | 1. Prevent Shock 2. Stabilize the women in shock 3. Reverse shock 4. Decrease blood loss 5. Compress blood vessels 6. Increase blood flow to vital organs 7. I don’t know |  |
| 206. | What is/are the indications for NASG application? (more than one answer is possible) | 1. Postpartum hemorrhage (PPH) 2. Shock due to ectopic pregnancy 3. Post cesarean hemorrhage 4. Shock due to trauma with injury/hemorrhage below the diaphragm 5. I don’t know |  |
| 207. | When NASG applied for women with obstetric haemorrhage management? (more than one answer is possible) | 1. Blood loss >750ml 2. Systolic blood pressure <90 mmHg 3. Pulse >110 bpm 4. I don’t know |  |

| **Part- II Knowledge on Non-pneumatic Anti-Shock Garment (NASG)** | | | |
| --- | --- | --- | --- |
| 201. | Have you ever heard about Non pneumatic Anti-Shock Garment (NASG)? | 1. Yes 2. No |  |
| 202. | What is the NASG looks like? | 1. Bottom half of suit 2. Gown 3. Trouser |  |
| 203. | What is your first source of information about NASG? | 1. Training 2. Health institution as they used management modality 3. College/ university education 4. Internet Other specify_________ |  |
| 204. | How many segments does NASG have? | 1. Four 2. Six 3. Eight 4. Nine |  |
| 205. | What is/are the function of NASG? (more than one answer is possible) | 1. Prevent Shock 2. Stabilize the women in shock 3. Reverse shock 4. Decrease blood loss 5. Compress blood vessels 6. Increase blood flow to vital organs 7. I don’t know |  |
| 206. | What is/are the indications for NASG application? (more than one answer is possible) | 1. Postpartum hemorrhage (PPH) 2. Shock due to ectopic pregnancy 3. Post cesarean hemorrhage 4. Shock due to trauma with injury/hemorrhage below the diaphragm 5. I don’t know |  |
| 207. | When NASG applied for women with obstetric haemorrhage management? (more than one answer is possible) | 1. Blood loss >750ml 2. Systolic blood pressure <90 mmHg 3. Pulse >110 bpm 4. I don’t know |  |
| 208. | What is/are contraindications for use of NASG?  (more than one answer is possible) | 1. Viable fetus in-utero 2. Pulmonary edema 3. Bleeding above the diaphragm 4. Congestive heart failure due to mitral stenosis 5. Dyspnea 6. I don’t know |  |
| 209. | How to apply NASG?  (more than one answer is possible) | 1. Start at ankle and proceed up to umbilicus 2. Start at umbilicus then proceed to ankle 3. Start at any segment |  |
| 210. | When to remove NASG? (more than one answer is possible) | 1. Estimated blood loss decreased to <50ml/hr 2. Hemoglobin level is >7 or hematocrit is >20% 3. Pulse <100bpm 4. Systolic BP 90mmHg or greater 5. The woman is conscious and aware 6. I don’t know |  |
| 211. | What does it mean the rule of 20 cautions for NASG removal? | 1. The time interval between removal of successive segments 2. Blood pressure and pulse which require reapply of segment/s when BP falls by 20 mmHg or pulse increases by 20 bpm respectively. |  |

| **Part III- Attitude towards Non-pneumatic Anti-Shock Garment (NASG)** | | |
| --- | --- | --- |
| 301. | 3The use of non-pneumatic anti shock garment is necessary for the management of obstetric haemorrhage management in all settings. | 1. Strongly disagree 2. Disagree 3. Neutral 4. Agree 5. Strongly agree |
| 302. | NASG can be used along with standard treatment protocols of obstetric haemorrhage management | 1. Strongly disagree 2. Disagree 3. Neutral 4. Agree 5. Strongly agree |
| 303. | NASG can be applied with minimum procedures in short period of time. | 1. Strongly disagree 2. Disagree 3. Neutral 4. Agree 5. Strongly agree |
| 304. | Removal NASG requires a lot of procedures that takes time. | 1. Strongly disagree 2. Disagree 3. Neutral 4. Agree 5. Strongly agree |
| 305. | Anti-shock garment is only beneficial to people in the rural areas/primary care settings. | 1. Strongly disagree 2. Disagree 3. Neutral 4. Agree 5. Strongly agree |
| 306. | Manual removal of placenta is possible with NASG in place. | 1. Strongly disagree 2. Disagree 3. Neutral 4. Agree 5. Strongly agree |
| 307. | Anti-shock garment is effective in patients with cervical lacerations. | 1. Strongly disagree 2. Disagree 3. Neutral 4. Agree 5. Strongly agree |
| 308. | The garment should be a must in every health care facility that has maternity service. | 1. Strongly disagree 2. Disagree 3. Neutral 4. Agree 5. Strongly agree |
| 309. | The garment is only meant to be applied by health care professionals | 1. Strongly disagree 2. Disagree 3. Neutral 4. Agree 5. Strongly agree |

| **Part- IV Utilization of Non-pneumatic Anti-Shock Garment (NASG)** | | | |
| --- | --- | --- | --- |
| 401. | Have you received training on the use NASG? | 1. Yes 2. No |  |
| 402. | Does NASG available in your health facility? | 1. Yes 2. No 3. I don’t know | 404 |
| 403. | If yes to Q 402, how many NASGs available in your health facility? | _____(in number) 1. I don’t know in number |  |
| 404. | Have you ever used NASG in the management of obstetric haemorrhage management? | 1. Yes 2. No |  |
| 405. | If no to Q 404, what is/are the reason/s for not utilize NASG for obstetric haemorrhage management? (more than one answer is possible) | 1. Availability of other method 2. Effective management of third stage of labor 3. Lack of experience 4. Don’t know about NASG 5. The garment is not available |  |
| 406. | If you know how to use it, will you use it? | 1. Yes 2. No |  |
| 407. | If it is available, will you use it? | 1. Yes 2. No |  |

**Thank you for your participation!!!**
